# The Role of Translation-Associated Proteins in p53 Modulation: Mechanisms and Implications

**DOI:** 10.3390/ijms26178164

**Published:** 2025-08-22

**Authors:** Daria Kałużyńska

**Affiliations:** Department of Systems Biology and Engineering, Silesian University of Technology, Akademicka 16, 44-100 Gliwice, Poland; daria.kaluzynska@polsl.pl

**Keywords:** p53, apoptosis, ribosomal proteins, nucleolar stress, translation, translation regulation

## Abstract

Translation is the final stage of protein synthesis and involves a broad range of proteins—from those directly participating in the process, such as initiation factors and ribosomal components, to those involved in post-translational regulation. Beyond their canonical functions, many of these proteins also influence key signaling pathways, including those regulating cellular stress responses and tumor suppression. This review explores the current knowledge of translation-associated proteins that modulate the tumor-suppressor protein p53. It highlights the roles of ribosomal proteins, stress arising from impaired ribosome biogenesis (nucleolar stress), and various translation-related factors in influencing p53 stability and activity. By integrating findings from diverse studies, this work provides insight into the intricate interplay between translation and p53 signaling, emphasizing its relevance for cellular homeostasis and stress adaptation.

## 1. Introduction: Translation and miRNA

Translation, the process by which proteins are synthesized from messenger RNA (mRNA), is a highly regulated and energy-intensive stage of gene expression. It plays a central role in maintaining cellular balance by supporting processes such as cell growth, differentiation, and responses to stress. Translation is regulated at several stages—initiation, elongation, and termination—and by various post-translational modifications.

In addition to proteins directly involved in the translation process, such as initiation or elongation factors, this process is regulated by many other proteins, such as mTOR pathway proteins [1], stress kinases, ribosomal proteins (RPs), and antigens. This review explores the interactions between p53 and translation-related proteins, focusing on their underlying mechanisms.

## 2. Ribosome Biogenesis and Its Role in Cellular Function

Ribosome biogenesis is a highly intricate and energy-demanding process, accounting for over 50% of a cell’s total energy consumption [2,3,4,5]. A mature ribosome consists of 80 ribosomal proteins and four ribosomal RNAs (rRNAs). The biogenesis process begins in the nucleus and culminates in the cytoplasm, where mature ribosomes are produced. Human ribosomes consist of a large 60S subunit (comprising 28S, 5S, and 5.8S rRNAs and 47 RPs) and a small 40S subunit (with 18S rRNA and 33 RPs) [6,7,8]. Most ribosome biogenesis occurs in the nucleolus, where RNA polymerase I transcribes rDNA (ribosomal DNA) to form 47S pre-rRNA, which contains sequences for 18S, 5.8S, and 28S rRNAs. RNA polymerase III transcribes 5S rRNA, which is then transported to the nucleolus, where it combines with RPs to form ribosomal subunits. Subsequently, the subunits are exported to the cytoplasm, where they mature and become competent to perform protein synthesis [9].

Disruptions in ribosome biogenesis, caused by transcriptional, translational, or transport-related errors, can lead to ribosomal stress, resulting in an imbalance and accumulation of ribosomal proteins. These proteins can migrate from the nucleolus to the nucleoplasm, influencing the regulation of other nuclear proteins. This phenomenon, known as nucleolar or ribosomal stress, often correlates with impaired ribosome biogenesis and has a significant impact on p53 activity. In contrast, p53 can negatively regulate ribosome biogenesis in certain scenarios.

The insights summarized by Golomb et al. emphasize the critical role of ribosomal stress in linking translational regulation with tumor suppression. Disruption of ribosome biogenesis triggers nucleolar stress, which interferes with the proper nuclear translocation of ribosomal proteins. As a result, excess ribosomal proteins accumulate in the cytoplasm, where they modulate signaling pathways—including those governing apoptosis—by stabilizing the tumor-suppressor protein p53. Most ribosomal proteins exert a positive regulatory effect on p53, although some may also attenuate its stability, indicating a context-dependent influence. Enhanced p53 activity under conditions of ribosomal stress frequently promotes apoptotic pathways, reinforcing the role of translational fidelity in safeguarding cellular homeostasis and preventing tumorigenesis [10].

## 3. Ribosomal Proteins and p53 Regulation

Numerous studies have shown a significant effect of ribosomal stress on p53 activity. There are many examples in the literature of the existence of p53 activation mechanisms resulting from the overexpression of many ribosomal proteins (described further below), which, under the influence of ribosomal stress, accumulate in the nucleoplasm, upregulating the level of p53.

Deisenroth and Zhang [11] emphasized the critical role of ribosomal proteins in the negative feedback loop between p53 and MDM2. Their study revealed that disruptions in rRNA synthesis alone can lead to the release of unbound ribosomal proteins, which interact with MDM2 and stabilize p53. This mechanism links nucleolar stress directly with p53 activation, without requiring full impairment of ribosome assembly. Importantly, changes in the levels of p53 and MDM2 can serve as markers for nucleolar stress.

The literature provides numerous examples (listed in this paper) of activation mechanisms triggered by overexpression of various ribosomal proteins that regulate the p53 levels [12]. Most ribosomal proteins regulate p53 levels by binding to the central acidic domain of the MDM2 protein, thereby inhibiting the zinc finger activity of the ubiquitin E3 ligase. This mechanism can be regulated by the naturally occurring point mutation MDM2C305F [13,14]. The presence of this mutation prevents most RPs from attaching to this region, while RPL5 retains this ability.

A clear relationship has been established between p53 activation and the presence of RPL5 and RPL11 [8,15]. RPL5 and RPL11 are distinctive RPs because they can together form a ribonucleoprotein complex with 5S rRNA (RPL5-RPL11-5S rRNA), which makes the RPs more stable [16,17]. The 5S RNP interacts with and regulates MDM2, particularly when ribosome production is impaired, leading to accumulation of the 5S RNP and its enhanced binding to MDM2 [16,18]. This pathway is essential for p53 activation in response to stresses such as defects in ribosome biogenesis, chemotherapeutic drugs, nutrient starvation, and oxidative stress. Unlike p53 activation by DNA damage, which occurs independently of the 5S RNP–MDM2 interaction, this pathway serves as a major regulatory mechanism. Interestingly, defects in small ribosomal subunit production can also activate p53 via the 5S RNP, even without significant changes in large ribosomal subunit levels [19].

In addition to RPL5 and RPL11, several other ribosomal proteins can influence the activity of p53. These include RPS2 [20], RPS3 [21,22], RPS7 [23,24], RPS14 [25,26], RPS15A [27,28,29,30], RPS25 [31,32,33], RPS26 [34], the RPS27 family [35,36,37,38,39], RPL4 [40,41,42], RPL6 [43,44], RPL22 [45], RPL23 [46,47,48], RPL24 [49], RPL26 [50,51,52,53,54], RPL32 [55], RPL37 [56], and MRPL41 [57,58].

**Figure 1:**
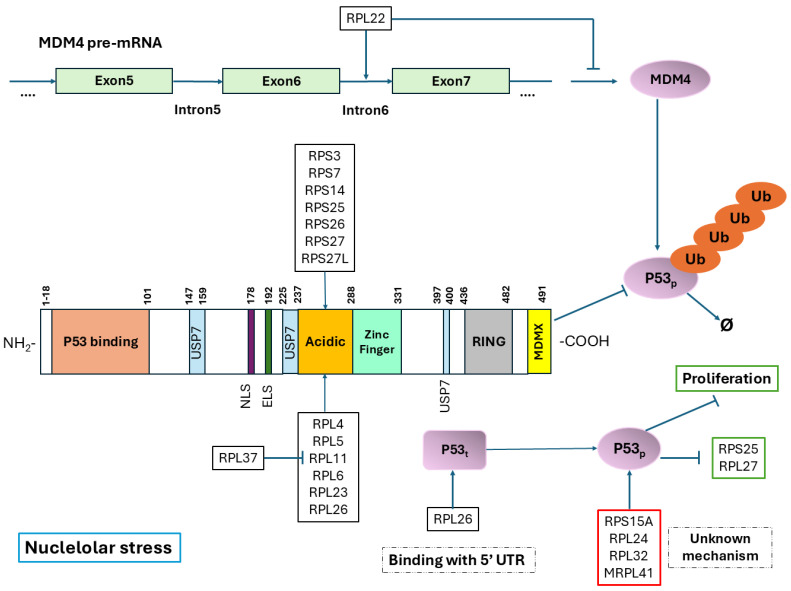
Diagram illustrating the mechanism of p53 activation in response to nucleolar stress. Most ribosomal proteins operate through a similar mechanism: they bind to the central acidic domain of MDM2, thus inhibiting its E3 ligase activity. RPS2 is unique among ribosomal proteins, as it binds to the RING domain of MDM2. Furthermore, both RPS3 and RPL26 are associated with the 5’UTR of the p53 transcript, enhancing its translation. The red box indicates proteins related to p53 activation, although the precise mechanism underlying this activation remains unknown. In the diagram, pointed tips represent positive regulation, while flat-ended arrows indicate negative regulation.

The following section explores the regulatory mechanisms of RP-dependent activation of p53. In addition, it discusses how p53 can influence the regulation of translation and ribosome biogenesis by targeting specific ribosomal proteins. A graph summarizing the relationships between p53 and ribosomal proteins is presented in Figure 1.

## 4. Regulation of p53 with Ribosomal Proteins

### 4.1. Small Ribosomal Subunit Proteins

RPS2 is one of the more recently identified ribosomal proteins involved in the regulation of the p53–MDM2 loop. In a 2020 study [20], immunoprecipitation assays using HEK293T cells demonstrated that RPS2 supports p53 activation by binding to MDM2 in its RING domain. The study also explored the regulatory relationship between RPS2 and MDM2, revealing that MDM2 targets RPS2 for ubiquitination. Furthermore, the findings showed that RPS2 influences p53 levels in a proteasome-dependent manner. Overexpression of RPS2 was found to stabilize p53, as indicated by the protein’s extended half-life. Furthermore, RPS2 induces the ubiquitination of MDM2, which, in turn, leads to the accumulation of p53. These findings highlight RPS2 as both a regulator and a substrate of MDM2.

RPS3 is another protein that modulates the negative feedback loop between p53 and MDM2. It binds to p53 and to the acidic domain of MDM2 through its KH domain, thus preventing ubiquitination of p53. This interaction is especially strong under oxidative stress. Knockdown experiments revealed that reducing RPS3 levels by 50% did not impair ribosome biogenesis; cytosolic RPS3 levels were affected without impacting ribosome-associated RPS3 [59]. Knockdown of RPS3 reduced p53 activity to 40% and disrupted one of MDM2’s functional domains [21].

The relationship between RPS7 and the p53-MDM2 loop is not as straightforward as initially assumed and involves many complexities. Beyond its regulation by binding to the MDM2 E3 ligase, RPS7 exhibits a particularly intriguing interaction with MDMX. Specifically, RPS7 inhibits MDM2 activity only when it is part of the MDMX complex. In HCT116 p53-/- cells, the knockdown of RPS7 resulted in a reduced half-life of MDM2, suggesting that RPS7 is a key protein for maintaining the stability of MDM2 under ribosomal stress. However, according to the study’s authors, this observation cannot be generalized due to inconclusive results in other cell lines, such as U2OS and SAOS-2. Furthermore, in the U2OS cell line, the ubiquitin-related protein RPS7-Ub loses its stabilizing properties for MDM2 and ceases to interact with MDMX, while still stabilizing the p53 protein [23,24].

The RPS14 protein inhibits p53 ubiquitination by binding to the central acidic domain of MDM2. In A549 cells, overexpression of RPS14 stabilizes endogenous p53 and prolongs its half-life [25]. RPS14 overexpression also induces p53-dependent cell-cycle arrest in the G1 and G2 phases. However, in p53-deficient cell lines (e.g., H1299 or HCT116 p53-/-), RPS14 does not affect the cell cycle. Ablation of RPS14 has been observed to diminish p53 activation induced by ribosomal stress. Interestingly, the removal of RPS14 can also activate p53 due to stress involving the RPL5/RPL11 complex.

RPS15A, although not extensively studied, has emerged as a significant marker in cancer biology. Studies indicate that the accumulation of RPS15A in tissues of colorectal cancer is correlated with tumor malignancy and a poor prognosis [27]. In vitro studies in six colon cancer cell lines revealed high expression of RPS15A. Knockout of RPS15A significantly inhibited cell proliferation, leading to the arrest of the p53-dependent cell cycle. Surprisingly, this increase in p53 levels did not induce apoptosis. Instead, the anti-apoptotic protein 14-3-3α was upregulated, mitigating apoptotic pathways. RPS15A has also been implicated in other cancers, including liver cancer, osteosarcoma, and lung cancer, highlighting its potential as an antitumor target. However, the precise mechanism for the regulation of p53 by RPS15A remains unexplored [27]. The work of [29] also demonstrated a relationship between RPS15A and p53 protein expression. In B-ALL cell lines (NALM-6, KOPN-8, RS4; 11 and BALL-1), where RPS15A is overexpressed, it was verified that silencing the RPS15A protein leads to ribosomal stress via activation of the p53 pathway. The work of [30] demonstrated an inverse relationship—in papillary thyroid cancer cells, the presence of RPS15A inhibits p53 protein expression, while RPS15A in a complex with γ-Glutamine cyclotransferase (GGCT) promotes the expression of this protein.

RPS25, a protein from the small ribosomal subunit, was first validated as a p53 regulator in 2013 [32]. It binds to the central domain of MDM2, forming a ternary complex with p53. RPS25 suppresses the E3 ligase activity of MDM2, thus inhibiting the ubiquitination of p53. Under ActD-induced ribosomal stress, p53 negatively regulates RPS25 transcription by binding directly to the S25 promoter, as shown in HCT116 wt and HCT116 p53-/- cell lines.

Later studies revealed that RPS26 overexpression stabilizes p53 and prolongs its half-life in HCT116 cells. Interestingly, RPS26 knockdown can also stabilize and activate p53, although this does not result in p53-induced transactivation of other proteins, despite an increase in MDM2 mRNA levels [34]. RPS26 is also known to self-regulate by inhibiting the splicing of its own pre-mRNA [60].

RPS27 and RPS27L, despite their nearly identical structures (differing by only three amino acids at the N-terminus [61]), exhibit different mechanisms of regulation but with similar effects. Proteins in the RPS27 family are among the first discovered ribosomal proteins regulated by p53. Specifically, the RPS27 gene is repressed by p53, while RPS27L is induced by it [35,36]. An interesting mechanism involves the targeting of both proteins for ubiquitination by binding to MDM2. This competition for MDM2 binding reduces the ability of p53 to interact with MDM2, thus preventing its ubiquitination. Under normal conditions, both proteins, unlike most other ribosomal proteins, accumulate primarily in the cytoplasm and only translocate to the nucleoplasm during stress. In SJSA and A549 cells, silencing of RPS27L destabilized p53. However, this effect was not consistent across all tested cell lines (e.g., it was absent in HCT116 and U2OS cells) [36].

Another member of this family, RPS27a, binds to MDM2 at a less common site: the zinc finger domain. Like RPS27, RPS27a is targeted by MDM2 for proteasomal degradation. Studies have shown that RPS27a is particularly sensitive to low doses of ActD, with a significant reduction in its level [62].

### 4.2. Large Ribosomal Subunit Proteins

Ribosomal proteins that are part of the large ribosomal subunit exhibit a more diverse pattern of p53 regulation than those of the small subunit. The literature indicates that the main mechanism of p53 protein regulation by RPS is blocking the activity of the MDM2 protein by binding to different domains. In turn, proteins of the large ribosomal subunit regulate p53 expression via other indirect mechanisms.

Research conducted in 2016 demonstrated that suppression of RPL4 in U2OS and H1299 cells induces ribosomal stress, triggering activation of p53 mediated by the RPL5/RPL11/MDM2 complex. In contrast, RPL4 overexpression also induces ribosomal stress, leading to activation of p53 and cell-cycle arrest [40]. However, the key difference between these scenarios is that RPL4 overexpression additionally inhibits MDM2 by binding to its central acidic domain. The work of [41,42] identified the proteins PRC1 (in ovarian tumor cells) and CCT8 (in colorectal cancer cells) as potential regulators of the p53 expression mechanism. These proteins compete by blocking access to the same binding domain to which the RPL4 protein attaches.

RPL6 presents an interesting case of a ribosomal protein that interacts with MDM2 in a negative feedback loop. Upon ribosomal stress, RPL6 translocates from the nucleolus to the nucleoplasm, where it binds to MDM2 [43]. In the H1299 p53 -/- cell line, RPL6 was found to positively influence MDM2 levels. However, in A549 cells, knockout of RPL6 resulted in a decrease in p53 protein levels. In HCT116 cells, RPL6 overexpression was shown to enhance MDM2-mediated stabilization of p53, significantly increasing the half-life of p53. Interestingly, RPL6 is also a substrate for the MDM2 ubiquitin ligase, suggesting that MDM2 negatively regulates RPL6 [43].

RPL22 is an interesting example of a ribosomal protein whose p53-regulating properties have been discovered recently. The work of [45] showed that RPL22 binds to intron 6 of MDM4 pre-mRNA, causing its inactivation through aberrant splicing (exon 6 is skipped and an incomplete form of MDM4 is created), which leads to reduced P53 ubiquitination.

RPL23 is one of the most well-studied p53 regulators among ribosomal proteins, along with RPL5 and RPL11. It regulates p53 by binding to MDM2 and inhibiting its E3 ligase activity [63]. RPL23 binds to MDM2 at the acidic domain. Experiments in U2OS cells demonstrated the formation of a complex comprising MDM2, RPL11, RPL5, and RPL23, suggesting that these proteins are independently attached to MDM2 [64]. Under stress induced by low doses of ActD, a drastic decrease in RPL23 levels was observed, while RPL11 levels remained unchanged [46].

RPL26 has been characterized as a direct positive regulator of p53. It binds to the 5′-UTR and 3′-UTR of the p53 mRNA, enhancing its translation [52,65]. However, RPL26 is negatively regulated by MDM2, which binds to RPL26 via its acidic domain, directing it toward ubiquitination and inhibiting its ability to stimulate p53 translation. In U2OS and MCF-7 cells with silenced MDM2, a twofold reduction in polyubiquitinated RPL26 was observed, highlighting the specificity of this interaction [12]. In particular, after ionizing radiation, RPL26 was found to bind to the 5′-UTR p53 mRNA, even in the presence of MDM2, which would typically hinder this interaction [50].

Ribosomal protein L37 is a very interesting protein. According to the work of [56], this protein indirectly inhibits p53 by negatively regulating RPL11. The exact mechanism of action has not been described. This relationship was described by examining U2OS GFP-L37 cells and silencing GFP with siRNA. After silencing, an increased level of expression of the RPL11 protein was observed, the role of which was described earlier.

The mitochondrial ribosomal protein MRPL41 induces p53-dependent apoptosis by promoting the translocation of p53 from the cytoplasm to the mitochondria. Although the exact mechanism behind this induction remains unclear, it has been speculated that MRPL41 may regulate other apoptotic effectors. However, there is currently no direct evidence to support this regulation [57,58].

## 5. Other Proteins Involved in Translation

**Figure 2:**
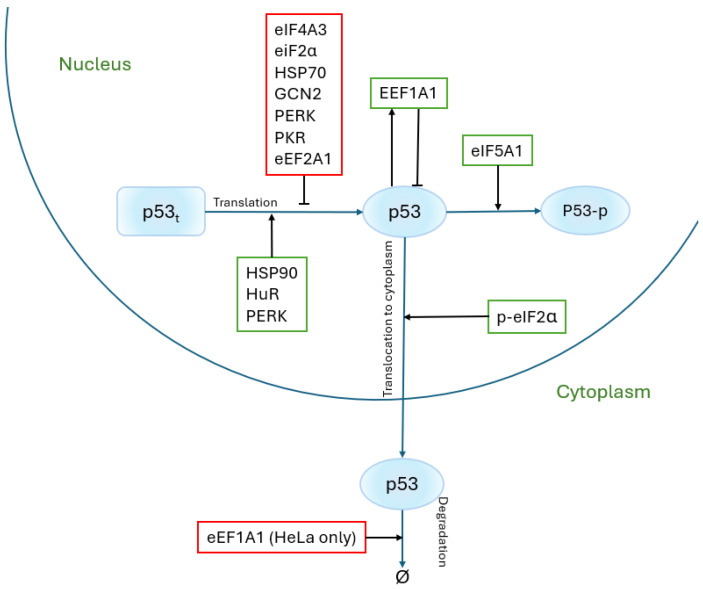
Diagram of interactions of other proteins involved in P53 regulation. Sharp arrowheads represent the promotion of protein synthesis, while flat arrowheads indicate its inhibition.

In addition to ribosomal proteins, many additional proteins play crucial roles in the translation process. These include factors involved in initiation, elongation, and termination, as well as regulatory proteins that influence these stages or participate in post-translational modifications. This section explores the proteins associated with translation, their contributions to the process, and their regulatory interactions with p53, a key tumor-suppressor protein. All proteins mentioned in the text and their relationships with the p53 protein are also presented (Figure 2).

Among eukaryotic initiation factors (eIFs), eIF2, eIF5A1, and eIF4A3 are particularly significant. eIF2 is responsible for delivering Met-tRNA to the 40S ribosomal subunit. This step is critical for the accurate selection of the start codon. eIF2, in combination with eIF5, facilitates the hydrolysis of GTP to GDP, allowing the proper assembly of the translation initiation complex [66]. Interestingly, eIF2 plays a dual role in the regulation of p53. In line with the findings of Pakos-Zebrucka et al. [67], phosphorylation of eIF2α impairs the exchange of GDP for GTP by eIF2B, thereby preventing the formation of the ternary complex eIF2–GTP–Met–tRNAi. This limits the delivery of the initiator tRNA to the 40S ribosomal subunit and leads to global translation attenuation. However, additional studies, such as those by Lee et al. [68], have shown that phosphorylated eIF2α (at Ser51) may reduce p53 stability and hinder its mitochondrial translocation under stress conditions. Conversely, the unphosphorylated form of eIF2α supports p53 stabilization.

eIF4A3, another initiation factor, is involved in RNA processing, including splicing and nonsense-mediated mRNA decay [69]. Studies using U2OS cells have shown that eIF4A3 can inhibit p53 expression, suggesting its potential role in the regulation of cell survival and apoptosis mechanisms. Given its involvement in essential RNA surveillance pathways, eIF4A3 is emerging as a significant node that connects translation and stress responses.

eIF5A1 stands out due to its multifaceted role in translation. Although traditionally associated with initiation, eIF5A1 also promotes elongation and termination [70]. In particular, eIF5A1 has been shown to upregulate p53 expression in various cellular contexts [71]. Research on aging cells has revealed that eIF5A1 maintains elevated translation rates, which are critical to maintaining proteostasis in senescent cells [72]. Further studies have demonstrated that increased eIF5A1 expression correlates with enhanced p53 phosphorylation at key residues (e.g., Ser15, Ser37, and Ser392), particularly under UV-induced stress in A549 and HCT116 cells [73].

Elongation factors (eEFs) are equally critical in translation and have been implicated in p53 regulation. eEF1A1 and eEF1A2 are responsible for delivering aminoacyl-tRNAs to the ribosome, ensuring the precise addition of amino acids to the growing polypeptide chain. However, both factors have been shown to negatively regulate p53 expression. For example, studies on U2OS and HeLa cells demonstrated that eEF1A1 suppresses p53 levels [74], while eEF1A2 exhibits similar effects in liver cells [75]. These findings suggest that elongation factors can act as suppressors of p53-dependent stress responses, potentially influencing cell-cycle progression and apoptosis. Another work [76] showed that eEF1A1 can form a negative feedback loop with p53.

Proteins such as HSP70 and HSP90 (Heat Shock Proteins) play critical roles in the proper folding of nascent polypeptides [77]. These molecular chaperones exhibit opposing effects on the regulation of p53. HSP70 destabilizes p53 by unfolding its tertiary structure, effectively inhibiting its tumor-suppressor functions. In contrast, HSP90 promotes ATP-dependent folding of p53, enhancing its stability and activity as a transcription factor [78]. This duality underscores the complex interplay between protein quality control mechanisms and p53 functionality.

The RNA-binding protein HuR (human antigen R) is essential for mRNA stability and splicing. HuR has been shown to directly interact with the 3′UTR of p53 mRNA, stabilizing it and promoting p53 expression [79,80]. Studies in RKO cells revealed that HuR enhances p53 levels in response to UVC-induced stress [81]. Similarly, silencing HuR in NIH3T3 cells reduced p53 expression, further emphasizing its role in p53 regulation [82].

GCN2 (General Control Nonderepressible 2), a kinase responsible for phosphorylating eIF2α, is a crucial regulator of translation during stress. By inhibiting general translation, GCN2 promotes the selective synthesis of stress-responsive proteins. Silencing GCN2 has been shown to activate p53, indicating its suppressive role in p53 translation [83,84]. Similarly, the eIF2α kinases PERK and PKR regulate translation under stress conditions. PERK activation can enhance p53 expression, depending on the cell type, as shown in CRL-2813 cells [85]. However, in MEF and HT1080 cells, PERK and PKR activation led to reduced p53 levels, highlighting context-dependent variability in their effects [86].

## 6. Conclusions

**Figure 3:**
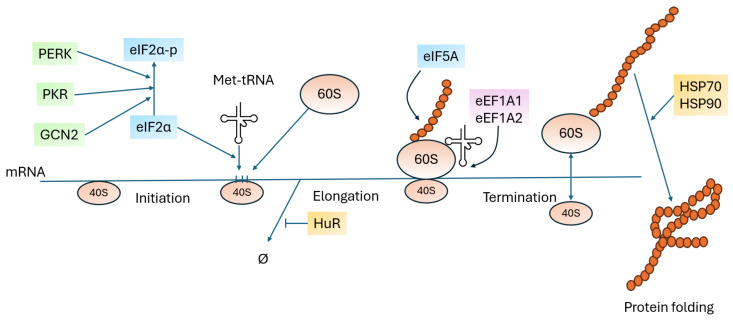
Simplified schematic of the translation process, highlighting the regulatory proteins involved in modulating p53 expression levels, as discussed in this paper.

Proteins involved in the translation process can also participate in the regulation of other cellular proteins. Using p53 as an example, it has been demonstrated that this regulation can occur bidirectionally. Translation-related proteins can promote or inhibit p53 synthesis, and, conversely, their expression and activity can be regulated by p53 itself. It has also been shown that proteins involved in each stage of the translation process regulate p53 expression (Figure 3).

This reciprocal relationship exemplifies the complexity of translational control mechanisms. Translation factors such as eIF2, eIF4A3, and eIF5A1 influence p53 synthesis by modulating specific stages of translation. For example, phosphorylated eIF2α (p-eIF2α) promotes the activation of p53 under cellular stress, enabling efficient stress-response mechanisms. In contrast, other proteins, such as eEF1A1 and eEF1A2, have been shown to suppress p53 levels, thus impacting its tumor-suppressing functions. This article also presented the important influence of ribosomal proteins on p53 expression. It was shown that many ribosomal proteins have a binding site for the acidic domain of the MDM2 protein, which acts as a negative regulator of p53. It was also shown that p53 can regulate the expression of ribosomal proteins (RPS25 and RPL27).

This bidirectional regulation highlights the interconnected nature of translation and cellular homeostasis. Disruptions in these processes, such as those caused by mutations or stress conditions, can lead to significant downstream effects, including altered protein synthesis, impaired stress responses, and cancer progression.
